# Supplementary material for: Skeletal muscle Nox4 knockout prevents and Nox2 knockout blunts loss of maximal diaphragm force in mice with heart failure with reduced ejection fraction
Source: Free Radic Biol Med. Author manuscript; Available in PMC 2024 Jan 1. (PMC10191720; doi:10.1016/j.freeradbiomed.2022.11.025)
Supplement: Supplement [file NIHMS1894160-supplement-Supplement.docx]

**
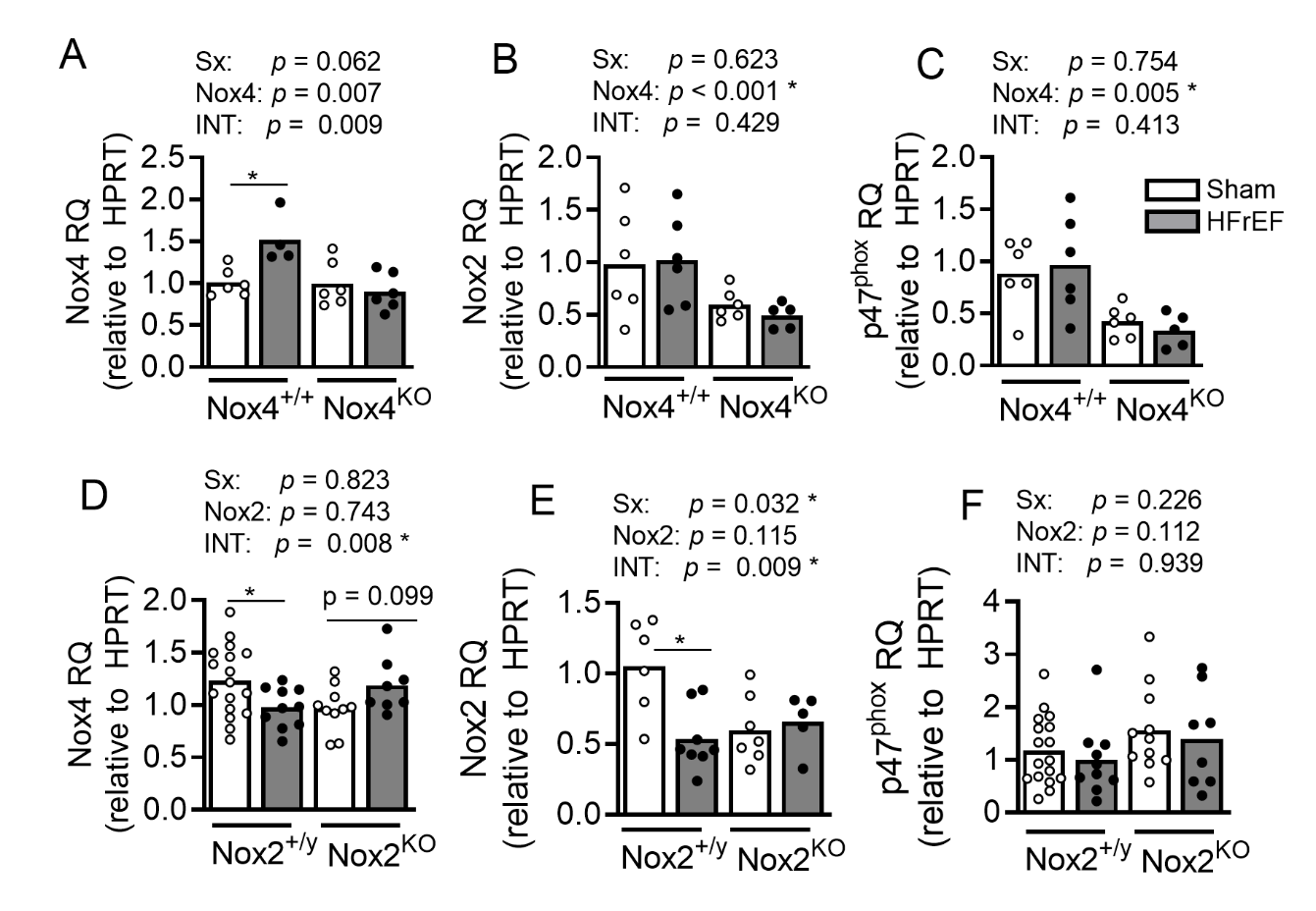
**

**Supplemental Fig. 1 –mRNA abundance of Nox subunits.** mRNA abundance of Nox4 (A,D), Nox2 (B, E), and p47^phox^ (C, F) measured from diaphragm muscle homogenates for the skmNox4 (A, B, C) and skmNox2 (D, E, F). Statistical analysis by two-way ANOVA with *p* values shown for effects of surgery (sham vs. HFrEF; Sx), Nox4 or Nox2, and the interaction of these two effects (Int). Bonferroni’s post-hoc test performed where appropriate. **p* < 0.05.

**Supplemental Table 1 – skmNox4 diaphragm contractile properties**

|  | SkmNox4^+/+^ | | SkmNox4KO | | *p* values | | |
| --- | --- | --- | --- | --- | --- | --- | --- |
|  | Sham (n = 7) | HFrEF (n = 7) | Sham (n = 7) | HFrEF (n = 6) | Surgery | Nox4 | Interaction |
| 300 Hz Specific  force (N/cm^2^) | 23.36 ± 1.49 | 18.60 ± 3.89 ^#^ | 23.95 ± 2.21 | 23.10 ± 2.51 ^$^ | 0.014 * | 0.033 * | 0.097 |
| 1 Hz Specific Force (N/cm^2^) | 3.70 ± 0.77 | 3.07 ± 0.65 | 4.07 ± 0.43 | 3.43 ± 0.77 | 0.040 * | 0.174 | 0.936 |
| 30 Hz Specific  force (N/cm^2^) | 5.61 ± 1.70 | 4.71 ± 1.27 | 6.18 ± 0.69 | 5.45 ± 1.93 | 0.174 | 0.274 | 0.887 |
| 50 Hz Specific  force (N/cm^2^) | 12.10 ± 2.47 | 9.76 ± 1.21 | 13.88 ± 1.70 | 12.32 ± 3.51 | 0.046 * | 0.028 * | 0.678 |
| Max Rate of  Contraction (% Max Force/ms) | 3.24 ± 0.40 | 3.14 ± 0.53 | 3.17 ± 0.33 | 2.99 ± 0.34 | 0.401 | 0.524 | 0.820 |
| Max Rate of  Relaxation (% Max Force/ms) | -5.23 ± 0.55 | -5.09 ± 0.58 | -5.12 ± 0.57 | -4.88 ± 0.50 | 0.409 | 0.495 | 0.829 |
| Time to peak  tension (ms) | 15.14 ± 1.21 | 14.50 ± 1.05 | 15.83 ± 0.75 | 14.83 ± 1.60 | 0.101 | 0.298 | 0.713 |
| ½ Relaxation Time (ms) | 14.26 ± 2.24 | 16.13 ± 3.30 | 14.37 ± 1.46 | 15.92 ± 1.88 | 0.080 | 0.957 | 0.865 |

Data are presented as mean ± SD**.** Statistical analysis by Two-way ANOVA with Bonferroni’s post-hoc test when appropriate. * *p* < 0.05. # p < 0.05 for Bonferonni multiple comparisons test relative to sham within strain. $ p < 0.05 compared to SkmNox4^+/+^ HFrEF.

**Supplemental Table 2 – skmNox2 diaphragm contractile properties**

|  | SkmNox2^+/y^ | | SkmNox2^KO^ | | *p* values | | |
| --- | --- | --- | --- | --- | --- | --- | --- |
|  | Sham (n = 15) | HFrEF (n = 12) | Sham (n = 13) | HFrEF (n = 9) | Surgery | Nox2 | Interaction |
| 300 Hz Specific  force (N/cm^2^) | 23.64 ± 1.41 | 19.05 ± 2.53 ^#^ | 23.37 ± 1.27 | 21.19 ± 1.36 ^#^ ^$^ | < 0.001 * | 0.046 * | 0.016 * |
| 1 Hz Specific  force (N/cm^2^) | 3.71 ± 0.47 | 3.19 ± 0.69 | 3.79 ± 0.59 | 2.97 ± 0.45 | < 0.001 * | 0.671 | 0.346 |
| 30 Hz Specific  force (N/cm^2^) | 5.73 ± 0.84 | 5.15 ± 1.29 | 5.82 ± 0.82 | 4.48 ± 0.76 | 0.001 * | 0.294 | 0.181 |
| 50 Hz Specific  force (N/cm^2^) | 12.88 ± 1.95 | 10.43 ± 1.88 | 13.42 ± 1.20 | 10.30 ± 1.97 | < 0.001 * | 0.686 | 0.518 |
| Max Rate of  Contraction (% Max Force/ms) | 3.07 ± 0.28 | 3.28 ± 0.54 | 3.09 ± 0.57 | 2.99 ± 0.29 | 0.939 | 0.563 | 0.174 |
| Max Rate of  Relaxation (% Max Force/ms) | -5.16 ± 0.35 | -4.97 ± 0.55 | -5.18 ± 0.36 | -5.14 ± 0.43 | 0.200 | 0.248 | 0.307 |
| Time to peak  tension (ms) | 15.13 ± 1.25 | 15.42 ± 2.57 | 15.92 ± 1.19 | 15.00 ± 1.87 | 0.535 | 0.717 | 0.245 |
| ½ Relaxation Time (ms) | 14.12 ± 1.83 | 15.25 ± 2.45 | 13.43 ± 1.34 | 14.35 ± 1.03 | 0.054 | 0.131 | 0.839 |

Data are presented as mean ± SD**.** Statistical analysis by Two-way ANOVA with Bonferroni’s post-hoc test when appropriate. * *p* < 0.05. # p < 0.05 for Bonferonni multiple comparisons test relative to sham within strain. $ p < 0.05 compared to SkmNox2^+/y^ HFrEF.

**
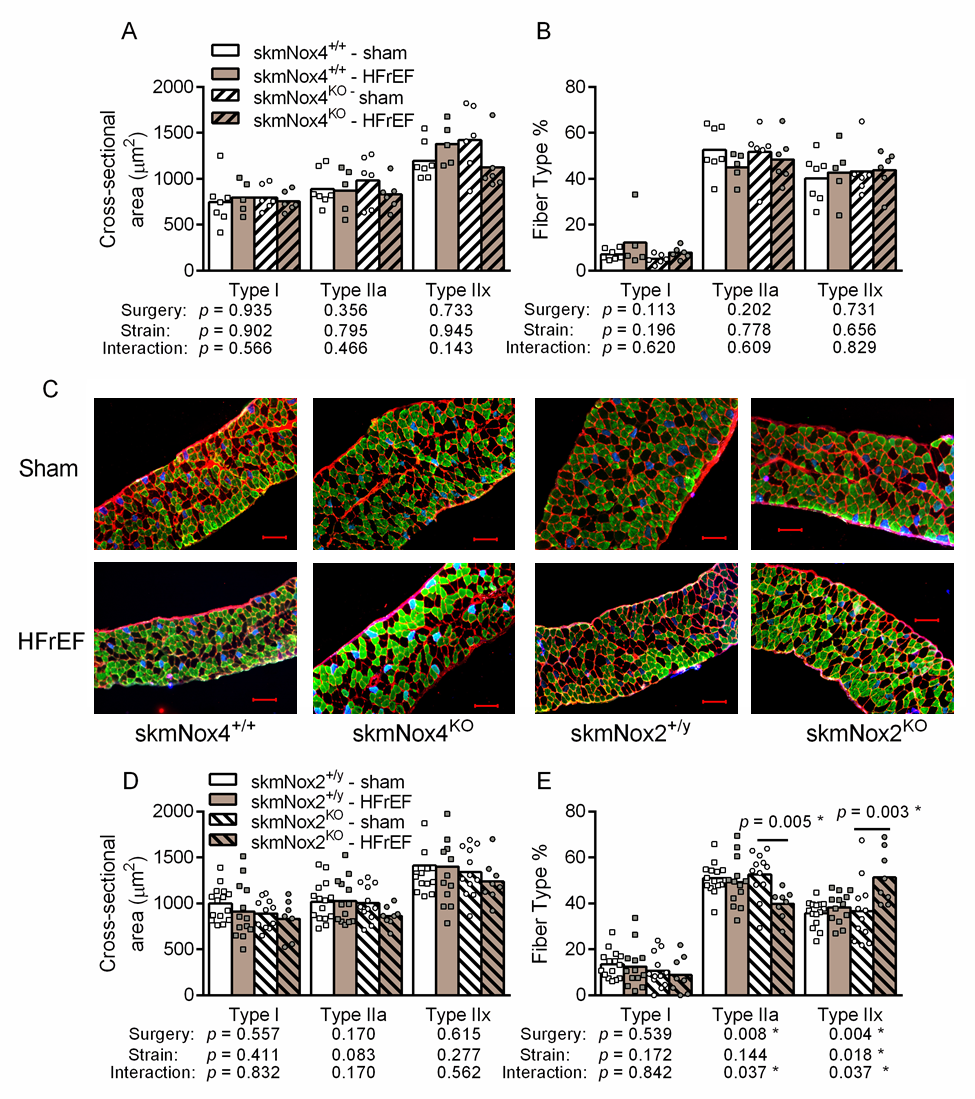
**

**Supplemental Fig. 2 – Diaphragm cross-sectional area and muscle fiber type distribution.** Fiber cross-sectional area by fiber type (A, C) and fiber type frequency (%) (B, D) from diaphragm micro sections. Scale bars represents 100 µM. Colors represent specific myosin heavy chain (MyHC) isoforms (blue = type I, green = type IIa, black = type IIb/x). Statistical analysis of cross-sectional area by linear mixed modeling. Statistical analysis of fiber type by two-way ANOVA with Bonferroni’s post-hoc test where appropriate. **p* < 0.05.

**
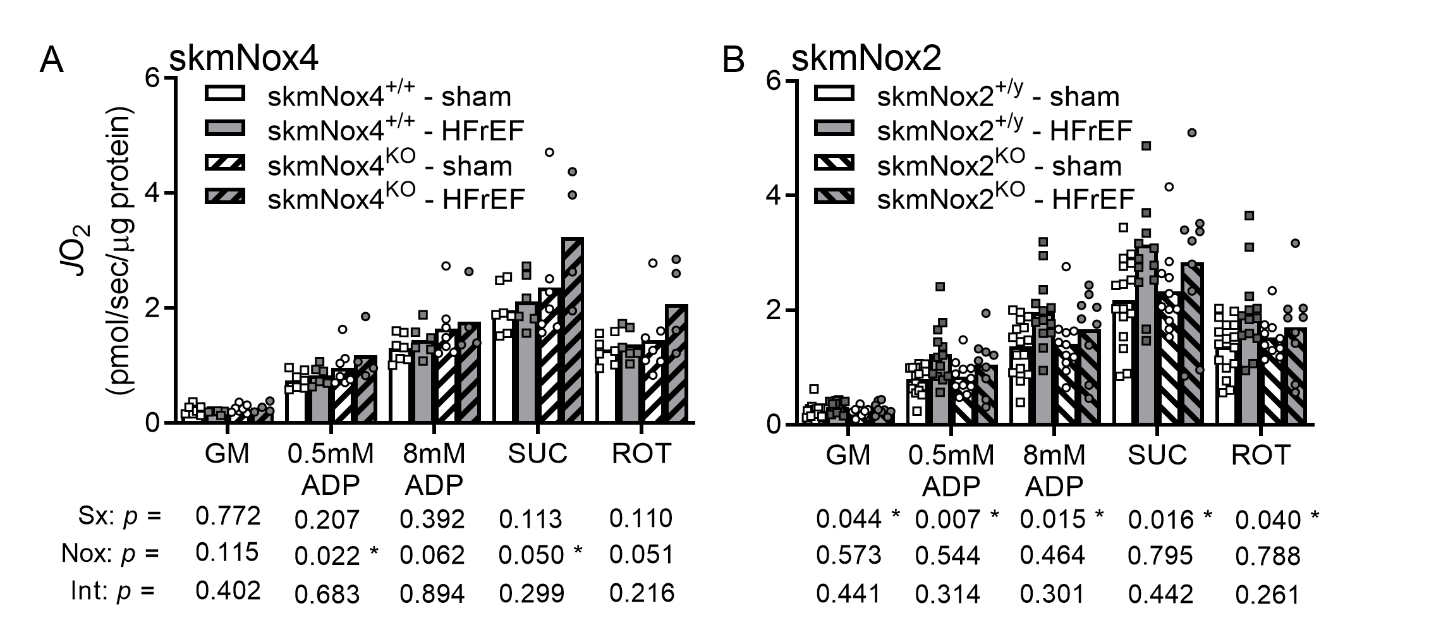
**

**Supplemental Fig. 3. Mitochondrial Respiration.** Rate of oxygen consumption (*J*O_2_) measured in saponin-permeabilized diaphragm bundles for (A) skmNox4 and (B) skmNox2 groups. Result normalized to fiber bundle protein content (*J*O_2_: pmol/sec/µg protein). Statistical analysis by two-way ANOVA for each substrate provided with *p* values shown for effects of surgery (sham vs. HFrEF; Sx), Nox4 or Nox2, and the interaction of these two effects (Int).**p* < 0.05

**Supplemental Fig. 4. Diaphragm mitochondrial protein abundance.** Representative membrane and gel images (A, C) and quantification for abundance of mitochondrial proteins (B, D) for skmNox4 (A, B) and skmNox2 (C, D) cohorts. Statistical analysis by two-way ANOVA. **p* < 0.05. CS: citrate synthase; CI-V: mitochondrial complex I-V.
